# Stabilizing Highly Active Ru Sites by Electron Reservoir in Acidic Oxygen Evolution

**DOI:** 10.3390/molecules29040785

**Published:** 2024-02-08

**Authors:** Jiayan Wu, Zhongjie Qiu, Jiaxi Zhang, Huiyu Song, Zhiming Cui, Li Du

**Affiliations:** Guangdong Provincial Key Laboratory of Fuel Cell Technology, School of Chemistry and Chemical Engineering, South China University of Technology, Guangzhou 510641, China; 202120123917@mail.scut.edu.cn (J.W.); 202221024255@mail.scut.edu.cn (Z.Q.); hysong@scut.edu.cn (H.S.); zmcui@scut.edu.cn (Z.C.)

**Keywords:** high-valence metal, ionic electronegativity, stabilized RuO_2_, acidic oxygen evolution reaction

## Abstract

Proton exchange membrane water electrolysis is hindered by the sluggish kinetics of the anodic oxygen evolution reaction. RuO_2_ is regarded as a promising alternative to IrO_2_ for the anode catalyst of proton exchange membrane water electrolyzers due to its superior activity and relatively lower cost compared to IrO_2_. However, the dissolution of Ru induced by its overoxidation under acidic oxygen evolution reaction (OER) conditions greatly hinders its durability. Herein, we developed a strategy for stabilizing RuO_2_ in acidic OER by the incorporation of high-valence metals with suitable ionic electronegativity. A molten salt method was employed to synthesize a series of high-valence metal-substituted RuO_2_ with large specific surface areas. The experimental results revealed that a high content of surface Ru^4+^ species promoted the OER intrinsic activity of high-valence doped RuO_2_. It was found that there was a linear relationship between the ratio of surface Ru^4+^/Ru^3+^ species and the ionic electronegativity of the dopant metals. By regulating the ratio of surface Ru^4+^/Ru^3+^ species, incorporating Re, with the highest ionic electronegativity, endowed Re_0.1_Ru_0.9_O_2_ with exceptional OER activity, exhibiting a low overpotential of 199 mV to reach 10 mA cm^−2^. More importantly, Re_0.1_Ru_0.9_O_2_ demonstrated outstanding stability at both 10 mA cm^−2^ (over 300 h) and 100 mA cm^−2^ (over 25 h). The characterization of post-stability Re_0.1_Ru_0.9_O_2_ revealed that Re promoted electron transfer to Ru, serving as an electron reservoir to mitigate excessive oxidation of Ru sites during the OER process and thus enhancing OER stability. We conclude that Re, with the highest ionic electronegativity, attracted a mass of electrons from Ru in the pre-catalyst and replenished electrons to Ru under the operating potential. This work spotlights an effective strategy for stabilizing cost-effective Ru-based catalysts for acidic OER.

## 1. Introduction

Hydrogen is one of the most promising energy carriers in the world and is considered as the ultimate energy in the 21st century with the advantages of high-mass energy density and environment-friendly energy [1]. Nowadays, hydrogen production mainly depends on fossil fuels, leading to global warming and severe air pollution [2]. In light of this, electricity-driven water splitting has great potential for hydrogen production because the electricity can be collected from intermittent renewable energy such as sunlight and wind [3]. There are three types of water electrolysis technologies: alkaline water electrolysis (AWE), proton exchange membrane water electrolysis (PEMWE), and solid oxide electrolysis cell (SOEC) [4]. Among these various water electrolysis technologies, proton exchange membrane water electrolysis attracts more attention due to its high purity (>99.9999 vol%) of hydrogen production, immediate response, and low ohmic losses [5].

Proton exchange membrane water electrolysis is an efficient approach for renewable hydrogen production to facilitate a carbon neutral future [6,7,8]. However, PEMWE stacks are usually carried at current densities exceeding 500 mA cm^−2^ [9]. To meet the need of faster proton transfer, the acidity close to the anode is stronger than 1 M H_2_SO_4_ when only using distilled water as the electrolyte, and this effect is aggravated when using acidic electrolytes or high current densities [10]. As a consequence, rare materials demonstrate desirable activity and stability at the anode of PEMWE due to the highly acidic and oxidative working conditions [11,12], where a sluggish oxygen evolution reaction (OER) involving four proton transfer steps takes place [5,13]. To date, IrO_2_ is currently the most widely used OER catalyst in acidic conditions, but its high cost (Ir: 140 USD g^−1^) [14] and scarcity have challenged its popularized application in PEMWE [15,16]. Due to the high overpotential of Ir in catalyzing the oxygen evolution reaction, Ir-based catalysts fail to meet the application requirements. RuO_2_, with relatively high earth abundance, lower price (Ru: 16 USD g^−1^) [14], and superior OER activity, is considered as a promising alternative to IrO_2_ [17,18]. Nevertheless, Ru-based catalysts suffer unavoidable overoxidation of Ru sites to form soluble RuO_4_ species at high overpotential, resulting in the loss of Ru active sites and degradation of OER performance [19,20,21]. Therefore, the stabilization of Ru species is pivotal for the development of highly active and cost-effective OER catalysts for PEMWE.

Formation of lattice oxygen vacancies (V_O_) and overoxidation of active Ru during acidic OER together accelerate RuO_2_ crystal structure collapse and surface Ru loss [21,22]. Given this, introducing electron-donating elements to stabilize low-charge Ru at fixed potential or improving the intrinsic activity of catalysts to decrease the operation potential at the controlled water-splitting current are two main methods [23]. Element doping has great potential to combine the advantages of the above two methods. Meanwhile, tuning the electron structure of Ru sites by foreign metal dopants has been considered as an effective strategy to hinder the dissolution of Ru [24,25,26]. Recently, many efforts have been aimed at improving the stability of Ru-based catalysts via high-valence metal substitution [24,27,28]. For example, Sun’s group reported that the incorporation of high-valence Nb in RuO_2_ weakened the covalency of the Ru–O bond with increased electron density around Ru sites, resulting in high OER stability (360 h at 200 mA cm^−2^) in acidic conditions [14]. Liu’s group developed a tungsten oxide matrix-confined Ru catalyst to stabilize low-valence Ru by facilitating electron transfer from oxidized W through the O bridge during an OER stability test (45 h at 10 mA cm^−2^) [29]. These studies demonstrated the impressive effect of stabilizing active Ru during OER by the modification of high-valence metal [30,31]. However, Zhang et al. revealed that the incorporation of different types of high-valence metals in RuO_2_ led to distinct OER activity and stability [27]. Therefore, the enhanced stability of these catalysts could be governed by underlying factors rather than solely relying on modification with high-valence metals (>+4). In a previous study, the ionic electronegativity of foreign metal dopants was correlated with the active site’s charge, which reflected the local metal–O bonding structure and the bonding strength [23]. On the other hand, Nørskov et al. reported that ‘stable’ RuO_2_ exhibited unsatisfactory catalytic activity due to the lack of unstable high-valence Ru^n>4+^ species [32,33]. In this consideration, the ionic electronegativity of the incorporated high-valence metals may be the deeper factor that affects the activity and stability of Ru. In addition, the role of ionic electronegativity in stabilizing the Ru site needs to be elucidated as well.

In this work, we introduced high-valence metals as electron reservoirs with different ionic electronegativities in rutile RuO_2_ to screen out a catalyst with remarkable activity and stability. We prepared the high-valence metal-substituted RuO_2_ using a modified molten salt method. The obtained catalysts exhibited a nanosheet morphology consisting of small nanoparticles with high surface areas, resulting in outstanding apparent activity of all samples. We correlated the ratio of surface Ru^4+^/Ru^3+^ species and the intrinsic activity and stability of the as-prepared catalysts with the ionic electronegativity of the incorporated cations. It was found that the substitution of high-valence metals with different ionic electronegativities could regulate the ratio of surface Ru^4+^/Ru^3+^ species where the oxygen evolution reaction takes place. RuO_2_ doped with the metal with the highest ionic electronegativity, Re, exhibited remarkable OER performance, characterized by a low overpotential of 199 mV, long-term stability exceeding 300 h at a current density of 10 mA cm^−2^, and even survived a 25 h operation at 100 mA cm^−2^ in a 0.1 M HClO_4_ electrolyte. We conclude that Re, with the highest ionic electronegativity, is equipped with a larger electron reservoir, which can serve as an electron accepter from Ru and induce high OER activity in pre-catalysts, and turn into an electron donor at high potential to inhibit the overoxidation of Ru during the OER process, according to the XPS results. Tuning the surface Ru^4+^/Ru^3+^ species content of rutile Ru-based oxides via the different ionic electronegativities of high-valence metal dopants is an effective strategy for designing high-performance Ru-based oxide OER catalysts in acidic electrolyte.

## 2. Results and Discussion

### 2.1. Characterization of the M_0.1_Ru_0.9_O_2_ Catalysts

**Figure 1:**
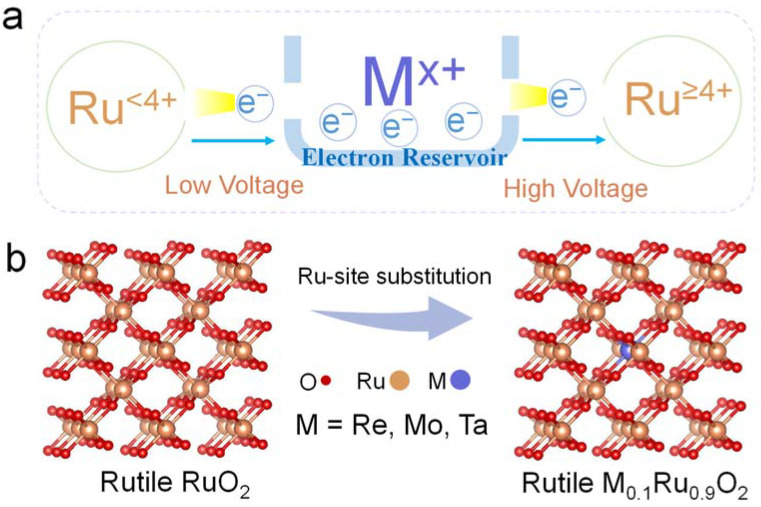
(**a**) Schematic diagram of the electron transfer process under different potentials. (**b**) Schematic image of partial substitution of Ru with Re, Mo, and Ta.

We are inspired by the fact that the ionic electronegativity of metal dopants can affect the Ru-O bonding nature in RuO_2_ [23]. In this work, we introduce high-valence metal dopants with different ionic electronegativities to serve as electron reservoirs in M-RuO_2_ to transfer electrons under different potentials (Figure 1a). As a proof of concept, Re^7+^, Mo^6+^, and Ta^5+^ with ionic electronegativities of 2.505, 2.101, and 1.925 were selected to partially substitute Ru in RuO_2_ [34]. To retain the rutile structure of the catalysts, the substituted amount was controlled at a small proportion of 0.1, which was the mole ratio of the foreign cation to the total metal in M-RuO_2_ (Figure 1b). High-valence metal (Re, Mo, Ta)-substituted RuO_2_ nanosheets (denoted as M_0.1_Ru_0.9_O_2_) were synthesized by a modified molten salt method. We used Brunauer–Emmett–Teller (BET) derived from N_2_ adsorption–desorption experiments to evaluate the specific surface area of the pre-catalysts. All homemade catalysts displayed high specific surface areas in the range from 154 m^2^ g^−1^ to 201 m^2^ g^−1^ (Figure S1), which was beneficial to the exposure of sufficient active sites to ensure high apparent activity. Typically, Re_0.1_Ru_0.9_O_2_ exhibited the highest BET surface area of 201 m^2^ g^−1^.

**Figure 2:**
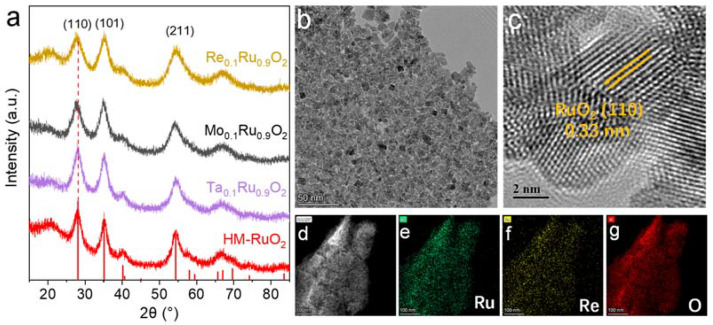
Morphological characterization of M_0.1_Ru_0.9_O_2_ (M = Re, Mo, Ta) and HM-RuO_2_. (**a**) XRD patterns, (**b**) TEM image, (**c**) magnified TEM image, and (**d**–**g**) TEM and corresponding elemental mapping images of Re_0.1_Ru_0.9_O_2_.

The X-ray diffraction (XRD) patterns (Figure 2a) showed that the M_0.1_Ru_0.9_O_2_ catalysts maintained the same crystal structure and phase as rutile RuO_2_ without any impurities. Three characteristic peaks at 28.1°, 35.3°, and 54.1° were attributed to the (110), (101), and (211) planes for rutile RuO_2_, respectively [35]. The (110) peak in the XRD spectra of M_0.1_Ru_0.9_O_2_ exhibited a negative shift compared to that of RuO_2_, which could be attributed to the larger radius of the incorporated M cation, resulting in lattice expansion (Figure S2). According to the transmission electron microscopy (TEM) analysis, Re_0.1_Ru_0.9_O_2_ presented a unique morphology of porous nanosheets assembled with ~5-nanometer particles (Figure 2b and Figure S3a). Such ultrasmall nanoparticle morphology favored the exposure of highly active sites to improve the OER activity. In addition, the rutile structure was also confirmed by high-resolution TEM, wherein the well-defined lattice fringes with interplanar distances of 0.33 nm were assigned to the (110) plane of the structure (Figure 2c) and displayed a broader lattice distance in contrast with homemade RuO_2_ (HM-RuO_2_). The expanded lattice distance in Re_0.1_Ru_0.9_O_2_ was consistent with the negative shift of the XRD peak (Figure S3d). Similar morphologies and crystal structures could be observed in HM-RuO_2_, Ta_0.1_Ru_0.9_O_2_, and Mo_0.1_Ru_0.9_O_2_ (Figures S3b–d, S4a–c, and S5a–c). These results indicated that high-valence metal substitution barely changed the morphology along with high-valence metals equipped into the rutile RuO_2_ lattice matrix. Moreover, TEM elemental mapping images presented uniform distributions of Ru, O, and high-valence foreign metals in the particles (Figure 2d–g, Figure S4d–g and S5d–g). The above results indicated the successful substitution of high-valence foreign metals in RuO_2_.

**Figure 3:**
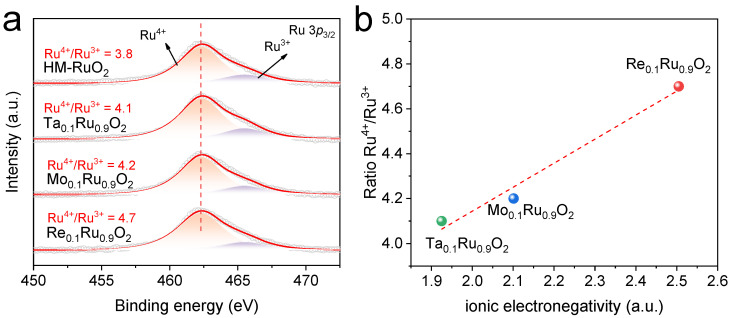
Electronic structures of M_0.1_Ru_0.9_O_2_ (M = Re, Mo, Ta) and HM-RuO_2_. (**a**) Ru 3*p* XPS spectra (The orange part indicates the content of Ru^4+^ and the purple part indicates the content of Ru^3+^). (**b**) Scaling between the ionic electronegativity [34] of foreign high-valence metals and the areal ratio of Ru^4+^/Ru^3+^ species.

The surface compositions and the corresponding electronic structures were further investigated by X-ray photoelectron spectroscopy (XPS), as the electrochemical reaction process takes place at the surface of the catalyst. As shown in Figure 3a and Table S1, the Ru 3*p*_3/2_ signals could be deconvoluted into two doublets. The two typical peaks of Ru 3*p*_3/2_ obtained for all samples were centered at 462.3 and 465.5, indexed to Ru^4+^ and Ru^3+^ species, respectively [14,36]. This result revealed that the oxidation state of Ru in the pre-catalysts was an Ru^4+^/Ru^3+^ mixture, which offered the potential for electron transfer. A linear relationship between the ratio of Ru^4+^/Ru^3+^ and ionic electronegativity was found, as shown in Figure 3b. The areal ratio of Ru^4+^/Ru^3+^ increased as the ionic electronegativity of the dopant metal increased from 1.925 (Ta^5+^) to 2.505 (Re^7+^), indicating the intensified valence state of Ru because the high-valence foreign metal with higher ionic electronegativity induced a reduced electron density in Ru. This trend of the variation in Ru valence was consistent with a previous report [23]. It was reported that Ru-based catalysts with a higher oxidation state of Ru exhibited higher OER activity due to optimized binding energy with oxygen intermediates [37]. In addition, the existence of high-valence foreign metals was also verified by their corresponding XPS spectra (Figure S6). According to previous reports, the oxidation states of Re, Mo, and Ta in the corresponding oxides were determined to be +7, +6, and +5, respectively, which were similar to our XPS results [38,39,40,41].

### 2.2. Acidic OER Performance

**Figure 4:**
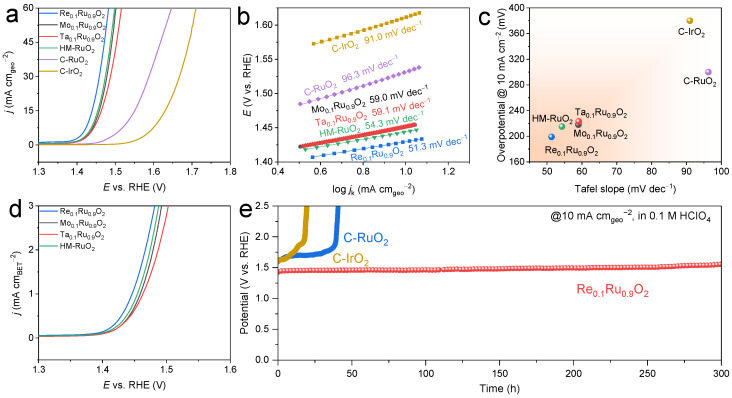
OER performance of the prepared electrocatalysts in 0.1 M HClO_4_. (**a**) LSV curves. (**b**) Tafel plots. (**c**) Comparison of overpotential and Tafel slope among M_0.1_Ru_0.9_O_2_ (M = Re, Mo, Ta), HM-RuO_2_, commercial RuO_2_, and commercial IrO_2_. (**d**) Specific activities of the prepared catalysts (OER currents normalized by BET surface area). (**e**) Chronopotentiometry tests of Re_0.1_Ru_0.9_O_2_, commercial RuO_2_, and commercial IrO_2_ at 10 mA cm^−2^.

The electrocatalytic performance of all catalysts for OER in 0.1 M HClO_4_ electrolyte was evaluated on a rotating disk electrode (RDE) in a typical three-electrode electrolytic cell. We used commercial RuO_2_ (denoted as C-RuO_2_) and commercial IrO_2_ (denoted as C-IrO_2_) as the benchmarks to investigate the effect of ionic electronegativity on acidic OER performance (Figure 4). It can be seen that the OER activities of all of the obtained catalysts far outperformed those of C-RuO_2_ and C-IrO_2_ (Figure 4a). Re-doped RuO_2_ with the best OER activity achieved a current density of 10 mA cm^−2^ with the lowest overpotential of 199 mV, while Mo- and Ta-substituted RuO_2_ catalysts delivered slightly larger overpotentials of 218 mV and 223 mV, respectively. Additionally, Re_0.1_Ru_0.9_O_2_ possessed the lowest Tafel slope of 51.3 mV dec^−1^ (Figure 4b,c), indicating that Re replacement accelerated the kinetics of the OER process. Electrochemical impedance spectroscopy (EIS) was also used to evaluate the OER kinetics. The obtained Nyquist plots were fitted using Autolab software “Nova 2.1.4 Version” (Figure S7), employing a consistent Randles equivalent circuit, including series and charge transfer resistance (*R*_s_ and *R*_ct_) as well as capacitance and phase elements [42,43]. It was found that Re-doped RuO_2_ exhibited the lowest charge transfer resistance with a small value of 16.0 Ω at an overpotential of 230 mV compared to Mo_0.1_Ru_0.9_O_2_ (19.6 Ω) and Ta_0.1_Ru_0.9_O_2_ (19.8 Ω), again implying its enhanced OER kinetics, which followed the same trend as the Tafel slope. The specific activities of the catalysts were determined by measuring their BET surface areas derived from nitrogen adsorption–desorption analysis. The ECSA-normalized OER activities were calculated to compare the intrinsic activities of M_0.1_Ru_0.9_O_2_. As can be seen in Figure 4d, the specific activities of those catalysts followed the order of Re_0.1_Ru_0.9_O_2_ > Mo_0.1_Ru_0.9_O_2_ > Ta_0.1_Ru_0.9_O_2_.

**Figure 5:**
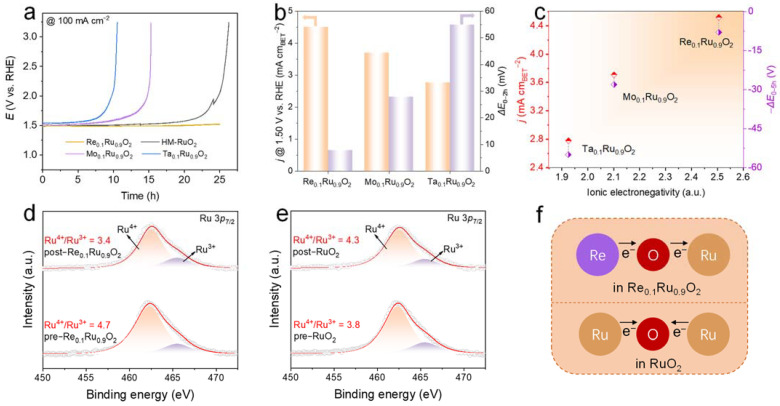
(**a**) Chronopotentiometry tests of M_0.1_Ru_0.9_O_2_ (M = Re, Mo, Ta, Ru) at 100 mA cm^−2^. (**b**,**c**) Comparison of specific activity (current density normalized by BET at 1.50 V vs. RHE) and potential variation at 100 mA cm^−2^ in a 2 h stability test as the ionic electronegativity increases. (**d**) XPS spectra of Re_0.1_Ru_0.9_O_2_ before and after the OER stability test. (**e**) XPS spectra of HM-RuO_2_ before and after the OER stability test. (The orange part indicates the content of Ru^4+^ and the purple part indicates the content of Ru^3+^). (**f**) Schematic diagram of electron transfer in Re_0.1_Ru_0.9_O_2_ and HM-RuO_2_ during the OER process.

Electrocatalytic stability is a more critical indicator for Ru-based catalysts. Chronopotentiometry (CP) tests were performed to investigate the OER durability of Re_0.1_Ru_0.9_O_2_, C-RuO_2_, and C-IrO_2_ in 0.1 M HClO_4_ at constant current densities of 10 mA cm^−2^ and 100 mA cm^−2^ (Figure 4e and Figure 5a). It is worth noting that Re_0.1_Ru_0.9_O_2_ exhibited highly desirable stability with a small degradation rate of 0.35 mV h^−1^ during 300 h continuous operation at 10 mA cm^−2^ and survived a 25 h CP test at 100 mA cm^−2^. Such stable performance was competitive with previously reported advanced Ru-based catalysts under similar conditions (Table S2). In contrast, commercial RuO_2_ suffered a sharp decline during the 50 h test at the current density of only 10 mA cm^−2^. Commercial IrO_2_ also failed to survive the long-term stability test at 10 mA cm^−2^. After the 300 h test, only 9.17 wt% Ru in Re_0.1_Ru_0.9_O_2_ was dissolved into the electrolyte, with a releasing rate of 0.015 μg_Ru_ h^−1^ based on ICP-MS analysis. The corresponding characterizations of post-stability Re_0.1_Ru_0.9_O_2_ were performed to observe the possible change during the OER test (Figure S8). The post-stability sample maintained the same morphology as that in Figure 2b. Further, the rutile crystal structure was preserved according to the HR-TEM image, which presented numerous lattice fringes indexed to the RuO_2_ (110) plane (Figure S8b). Moreover, the element compositions were still uniformly distributed, as seen in Figure S8c–f. The above results indicated that the Re_0.1_Ru_0.9_O_2_ catalyst maintained the bulk crystal structure well, suggesting the high acid corrosion resistance of Re_0.1_Ru_0.9_O_2_.

### 2.3. Investigation of Relationship between OER Stability and Electron Structure

Obtaining insights into the noticeable stability is significant for practical applications. It has been reported that high-valence metals with multiple oxidation states enable variations in Ru valence under different potentials. That is, the role of the high-valence metals in M_0.1_Ru_0.9_O_2_ can transform from electron receptors at on-site potential to electron donors at high potential [31,44]. According to the XPS results, dopants with high ionic electronegativity attracted a mass of electrons from Ru in the pre-catalysts and then generated active high-valence Ru species. The prepared M_0.1_Ru_0.9_O_2_ catalysts possessed high intrinsic activity compared to commercial RuO_2_, which helped to reduce the working potential at the controlled current density. The stability at a high current density of 100 mA cm^−2^ for the M_0.1_Ru_0.9_O_2_ catalysts was assessed to compare their acid tolerance at a relatively high potential (Figure 5a). The acid resistance of Re-doped RuO_2_ was superior to that of the homemade remainder. We increased the operation potential to 100 mA cm^−2^ in 2 h to quantify the acid tolerance at relatively high potential. Both the specific activity and the acid tolerance of high-valence metal-doped RuO_2_ improved as the ionic electronegativity increased (Figure 5b,c). XPS analysis of the post-catalysts after a 2 h stability test at 100 mA cm^−2^ was carried out to explore the variations in the ratio of surface Ru species (Figure 5d,e and Table S3). The ratio of Ru^4+^/Ru^3+^ decreased from 4.7 to 3.4 for Re_0.1_Ru_0.9_O_2_, while the ratio of Ru^4+^/Ru^3+^ increased from 3.8 to 4.3 for HM-RuO_2_, implying that electrons were transferred from Re with high ionic electronegativity to Ru during the OER process (Figure 5f). Consequently, introducing Re with high ionic electronegativity as an electron reservoir into RuO_2_ made a great contribution to the enhancement of OER stability.

## 3. Materials and Methods

### 3.1. Chemicals

Sodium nitrate (NaNO_3_), ruthenium (III) chloride hydrate (RuCl_3_·3H_2_O), molybdenum pentachloride (MoCl_5_), and tantalum (V) chloride (TaCl_5_) were purchased from Macklin Ltd. Commercial RuO_2_, commercial IrO_2_, and sodium perrhenate (NaReO_4_) were purchased from Energy Chemical Ltd. The 5 wt% Nafion^®^ ionomer was purchased from DuPont Co. All of the reagents and chemicals were used as received without further purification.

### 3.2. Preparation of M_0.1_Ru_0.9_O_2_ Catalysts

A family of M_0.1_Ru_0.9_O_2_ (M = Re, Ta, Mo, Ru) catalysts was synthesized by a modified molten salt method [45]. Generally, 0.45 mmol RuCl_3_, 0.05 mmol NaReO_4_, and 10 g NaNO_3_ powder were ground evenly in a mortar, and then the mixed powder was transferred into a 50 mL porcelain crucible. After the precursor salts were melted in a muffle furnace at 350 °C for 2 h, the catalysts were obtained in the flowing black molten salt. The mixture was removed for quick cooling to room temperature. The obtained product was rinsed with Milli-Q ultra-pure water thrice and dried in an oven at 60 °C. Taking HM-RuO_2_ samples as reference, 0.45 mmol RuCl_3_ and 10 g NaNO_3_ powder were ground evenly in a mortar. Other samples were prepared by the same procedure, except the foreign M salt was replaced with the corresponding precursor salts (TaCl_5_ and MoCl_5_).

### 3.3. Physical Characterization

Nitrogen adsorption–desorption analysis was carried out on a Tristar II 3020 gas adsorption analyzer (Micromeritics, Atlanta, GA, USA) to obtain the specific surface area of samples. X-ray diffraction (XRD) patterns were obtained from a Miniflex-600 powder diffractometer (Rigaku, Shibuya-ku, Tokyo, Japan) with Cu Kα radiation to determine the crystal structure of samples. The data were collected with a step size of 0.01 s and a dwell time of 0.1 s. Transmission electron microscopy (TEM) and the corresponding energy-dispersive X-ray analysis (EDX) were conducted using a Talos F200x field emission microscope (Thermo Fisher, Waltham, MA, USA) to detect the morphology and the element distribution of the samples. X-ray photoelectron spectroscopy (XPS) results were collected from a K-Alpha^+^ photoelectron spectrometer (Thermo Fisher Scientific) to analyze the surface composition and the valence of elements in the samples. All binding energies of the elements were referred to the C 1 s peak at 284.8 eV. Inductively coupled plasma (ICP) measurements were completed on an IRIS Intrepid instrument (Thermo Fisher, Waltham, MA, USA) to detect the content of the leaching element in the electrolyte after a long-term stability test. The moles of dissolved Ru (n_Ru_) in Re_0.1_Ru_0.9_O_2_ were determined by: n_Ru_ = m_Ru_/M_Ru_, where m_Ru_ is the mass of dissolved Ru after a 300 h OER stability test, and M_Ru_ is the molar mass of Ru (101.07 g mol^−1^).

### 3.4. Electrochemical Measurements

The electrochemical performance of the as-prepared catalysts was evaluated on a WaveDriver electrochemical instrument using a standard three-electrode system in 0.1 M HClO_4_, with the working electrode rotating at a speed of 2500 rpm and a graphite rod (diameter = 6 mm) serving as the counter electrode. Ag/AgCl (saturated KCl) was used as the reference electrode and catalyst-supported glassy carbon (GC, diameter = 5 mm) was regarded as the working electrode. All of the samples were measured based on independent tests. Typically, 5 mg catalyst powder, 5 mg carbon powder (XC-72), and 50 μL 5% Nafion were dispersed in 950 μL isopropanol and then sonicated for 1 h to form a homogeneous ink. Carbon powder served to increase the conductivity for the oxide catalysts. A 10 μL sample of the catalyst dispersion was dropped onto the glassy carbon electrode (0.25 mg cm^−1^). All potentials, except the potential of the stability tests, were calibrated on glassy carbon versus the reversible hydrogen electrode (RHE), using the following equation:

*E* (vs.RHE) = *E* (vs. Ag/AgCl) + 0.26 V

where 0.26 V is the potential difference between the Ag/AgCl reference electrode and RHE in 0.1 M HClO_4_ and calibrated in H_2_-saturated 0.1 M HClO_4_ prior to electrocatalytic measurement.

The linear sweep voltammetry (LSV) curves of all catalysts were collected in 0.1 M HClO_4_ within the potential range of 1.0~1.8 V vs. Ag/AgCl at a scanning rate of 10 mV s^−1^. Electrochemical impedance spectroscopy (EIS) analysis was conducted at 1.2 V vs. Ag/AgCl over the frequency range of 0.1~100 kHz. The obtained electrochemical data with iR compensation were denoted as in the following equation: *E*_correction_ = *E* − *iR*_*solution*_, where R_solution_ is the solution resistance from the EIS measurements. The stability tests were carried out on 0.5 cm × 0.5 cm carbon paper with a mass loading of 1 mg_oxide_ cm^−2^ under 0.1 M HClO_4_ at 10 mA cm^−2^ or 100 mA cm^−2^. The potentials of the stability tests were referenced to the RHE by using pure hydrogen calibration and corrected with iR loss on carbon paper.

## 4. Conclusions

In summary, high-valence metal dopants with high ionic electronegativity were employed as electron reservoirs to stabilize the Ru sites in Ru-based oxide lattice matrices for an efficient acidic OER process. A molten-salt method was used to synthesize the rutile RuO_2_ catalysts doped by M (Re, Ta, Mo) cations with different ionic electronegativities. We correlated ionic electronegativity with the ratio of surface Ru^4+^/Ru^3+^ species, the intrinsic activity, and the durability in acid of the catalysts. The high ionic electronegativities of the dopants induced high ratios of surface Ru^4+^/Ru^3+^ species, along with enhancements in both the intrinsic activity and the durability of the catalysts. Among all of the studied catalysts, Re_0.1_Ru_0.9_O_2_ exhibited excellent OER performance with a low overpotential of 199 mV and a negligible decay over a 300 h stability test at 10 mA cm^−2^. It was found that Re, with the highest ionic electronegativity, served as an electron donor in Re_0.1_Ru_0.9_O_2_ to inhibit the overoxidation of Ru at high potential and thus stabilize active Ru species. This work provides insight into the role of substitution with a high-valence metal with high ionic electronegativity as an electron reservoir to improve the stability of Ru-based materials.

## Data Availability

The data presented in this study are available in article and Supplementary Materials.

## Supplementary Materials

The following supporting information can be downloaded at: https://www.mdpi.com/article/10.3390/molecules29040785/s1. References [46,47,48,49,50,51,52,53] are cite in the Supplementary Materials.
