# Supplementary material for: Stabilizing Highly Active Ru Sites by Electron Reservoir in Acidic Oxygen Evolution
Source: Molecules. 2024 Feb 8;29(4):785. doi: 10.3390/molecules29040785 (PMC10892467; doi:10.3390/molecules29040785)
Supplement: Supplementary file 1 [file molecules-29-00785-s001.zip › molecules-2850245-supplementary.pdf]

Supporting Information

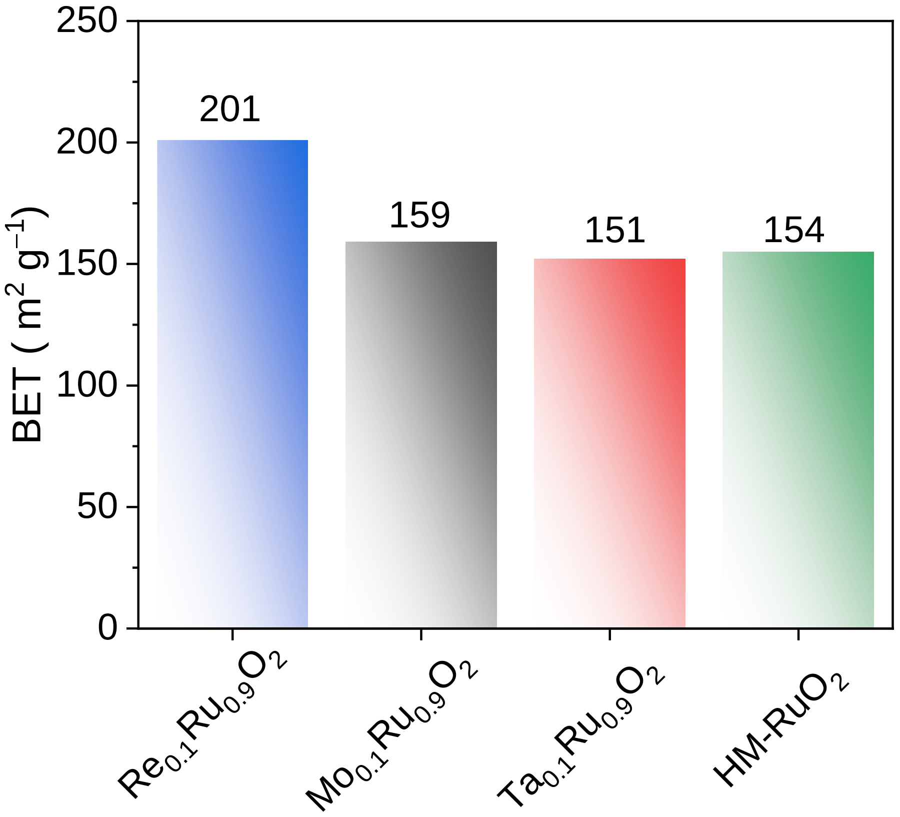

Figure S1. Surface area derived from BET.

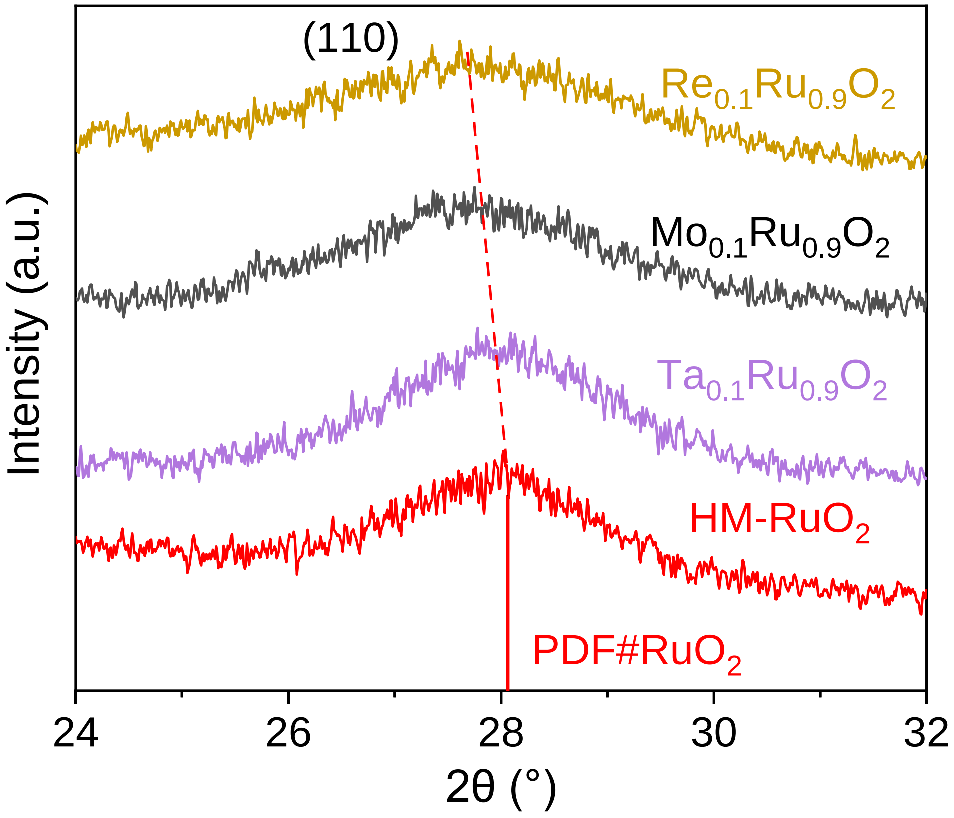

Figure S2. Magnified XRD patterns.

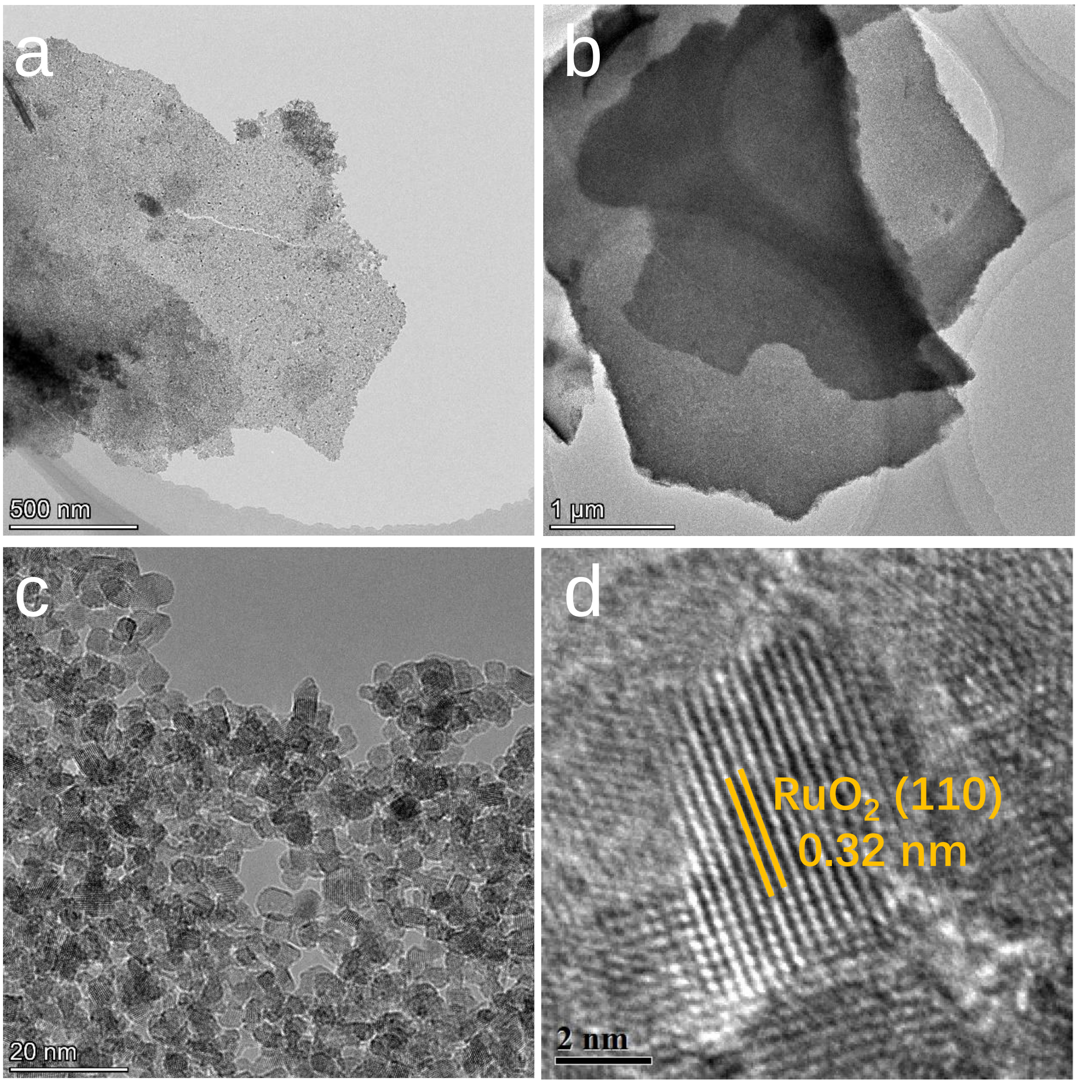

Figure S3. TEM images of (a) $Re_{0.1}Ru_{0.9}O_2$ and (b-d) HM-RuO_2.

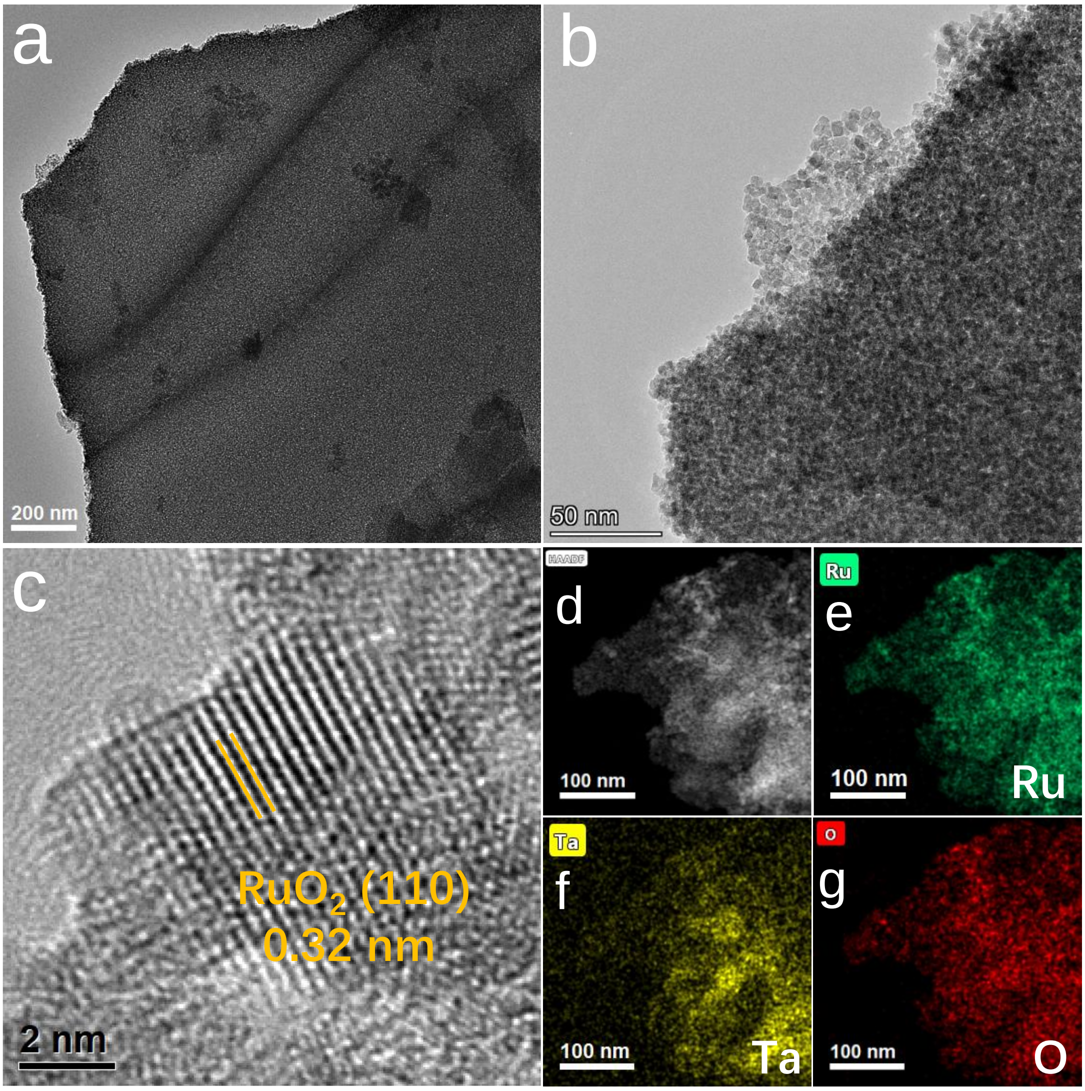

Figure S4. Morphology Structure of $Ta_{0.1}Ru_{0.9}O_2$. (a,b) TEM image, (c) magnified TEM image and (d–g) element mapping images.

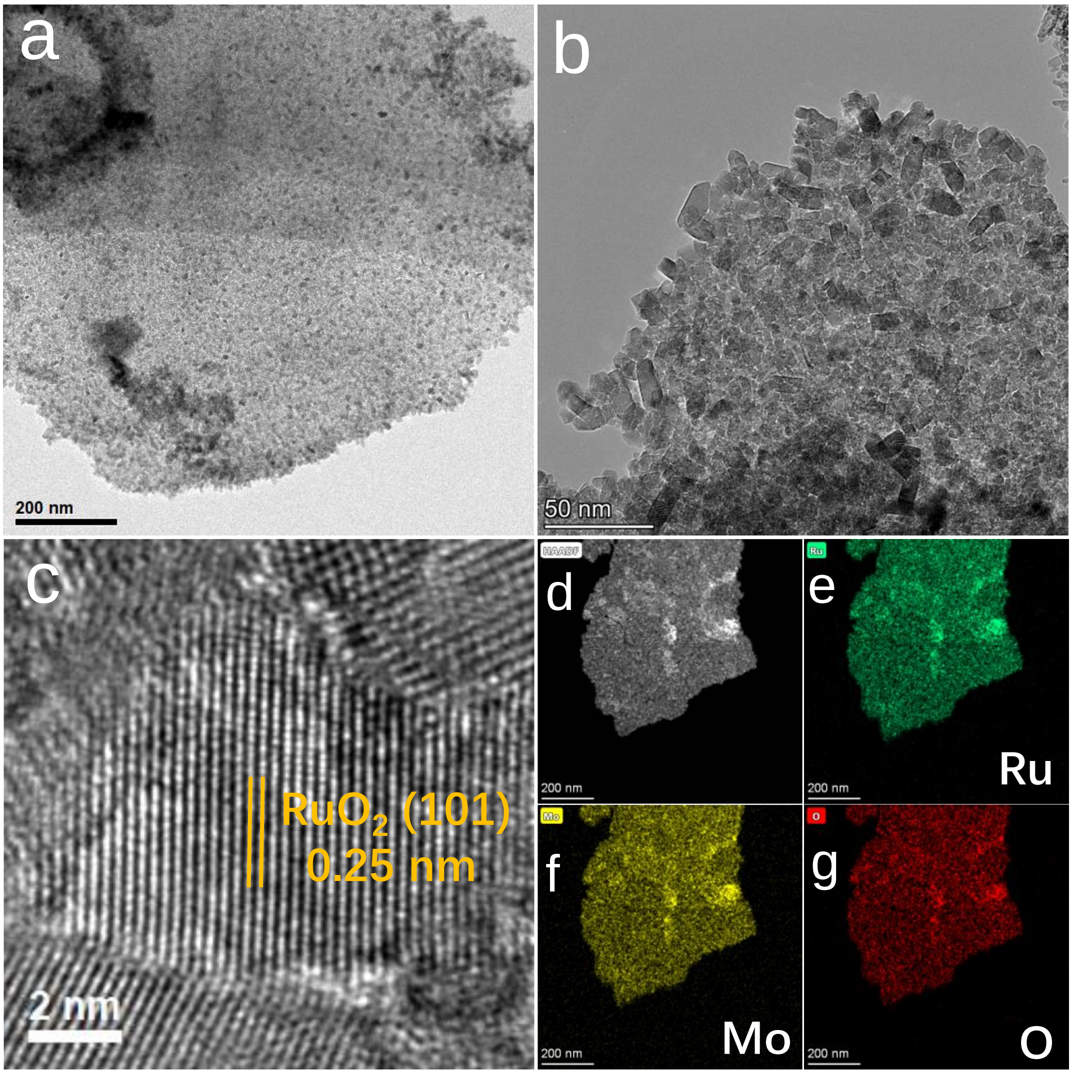

Figure S5. Morphology Structure of $Mo_{0.1}Ru_{0.9}O_2$. (a,b) TEM image, c) magnified TEM image and (d-g) element mapping images.

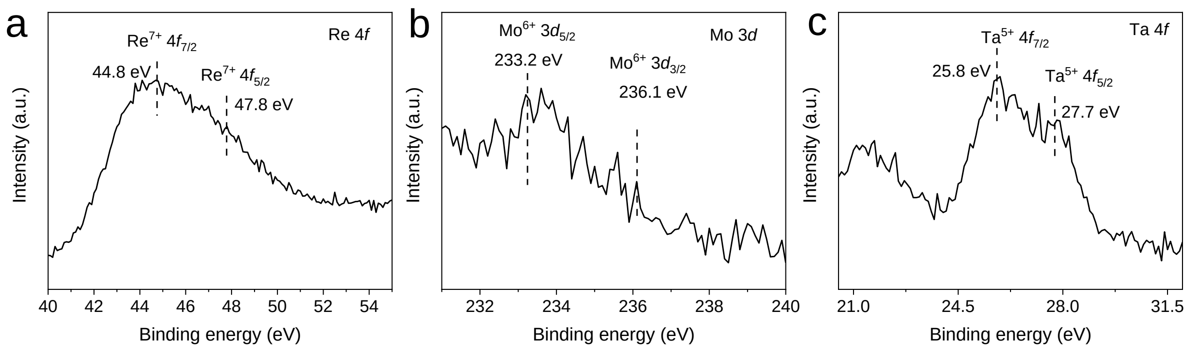

Figure S6. (a) Re 4*f* XPS spetrum of $Re_{0.1}Ru_{0.9}O_2$. (b) Mo 3*d* XPS spetrum of $Mo_{0.1}Ru_{0.9}O_2$. (c) Ta 4*f* XPS spetrum of $Ta_{0.1}Ru_{0.9}O_2$.

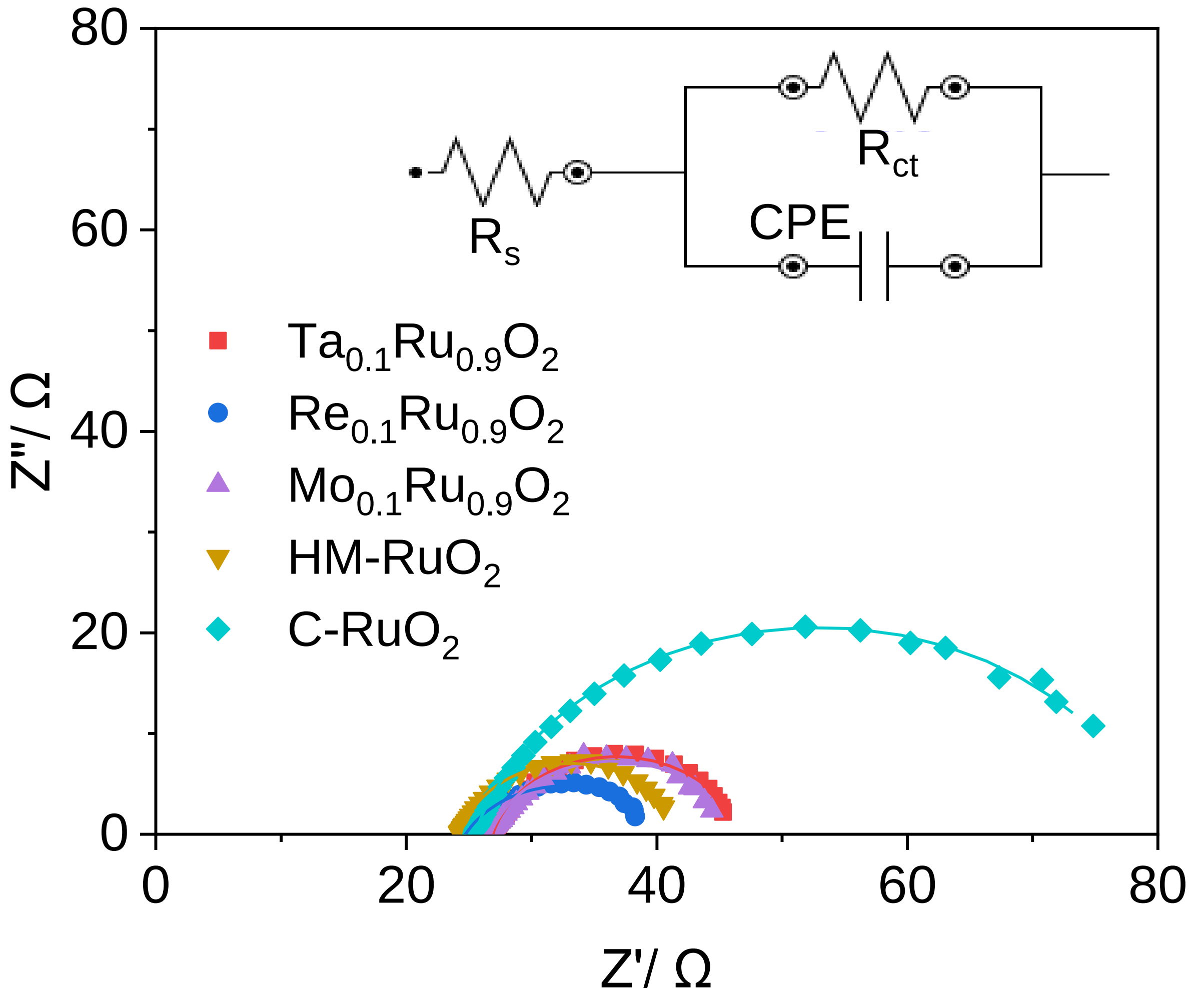

Figure S7. Nyquist impedance plot with fitted Randles circuit of $M_{0.1}Ru_{0.9}O_2$ (A = Re, Mo, Ta, Ru) and C-RuO_2. The obtained solution resistance was used for *iR* correction in electrocatalytic measurements at overpotential of 230 mV.

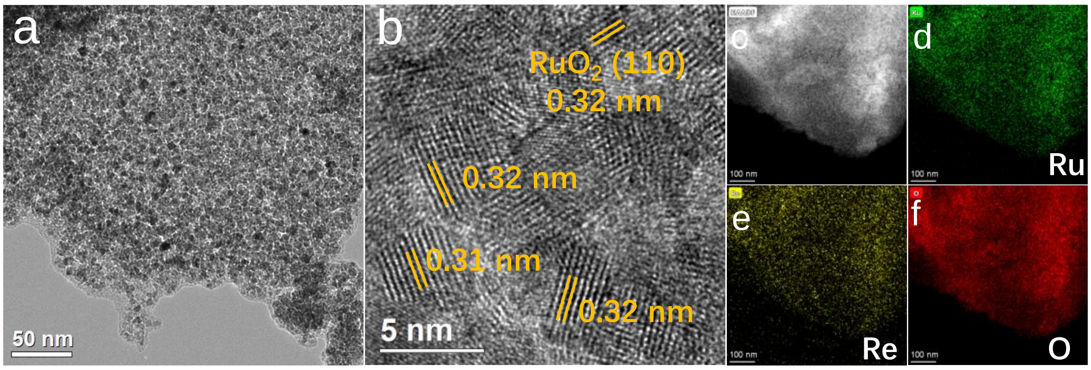

Figure S8. Structure characterizations for the post-stability $Re_{0.1}Ru_{0.9}O_2$. (a) TEM image. (b) HR-TEM image. (c–f) TEM and corresponding elemental mapping images.

Table S1. The fitting results of Ru 3$p_{3/2}$ spectra from XPS of pre-catalysts.

samples	Ru^{4+}		Ru^{3+}		Ru^{4+}/Ru^{3+}
	Position/eV	Area	Position/eV	Area	
$Re_{0.1}Ru_{0.9}O_2$	462.3	115618.6	465.5	24618.6	4.7
$Mo_{0.1}Ru_{0.9}O_2$	462.3	108228.1	465.5	25768.09	4.2
$Ta_{0.1}Ru_{0.9}O_2$	462.3	120228.1	465.5	29241.47	4.1
HM–RuO_2	462.3	105767.4	465.5	27767.4	3.8

Table S2. Comparison of OER performance for $Re_{0.1}Ru_{0.9}O_2$ with some benchmark Ru-based oxides under acidic media.

Catalyst	Electrolyte solution	Overpotential (mV)	Stability	Loading amount (mg cm^{-2})	Ref.
La-RuO_2	0.5 M H_2SO_4	η_{10} = 208	28 h (10 mA cm^{-2})	1.0 (Carbon Paper)	[1]
Si-RuO_x@C	0.5 M H_2SO_4	η_{10} = 220	100 h (10 mA cm^{-2})	1.6 (Carbon Paper)	[2]
RuO_2-WC NPs	0.5 M H_2SO_4	η_{10} = 347	10 h (10 mA cm^{-2})	0.51 (GC)	[3]
Ni-RuO_2	0.1 M $HClO_4$	η_{10} = 214	200 h	0.4 (GC)	[4]
$Ru_{0.85}Zn_{0.15}O_{2-\delta}$	0.5 M H_2SO_4	η_{10} = 190	50 h (10 mA cm^{-2})	0.416 (GC)	[5]
Ru/Se-RuO_2	0.5 M H_2SO_4	η_{10} = 190	24 h (10 mA cm^{-2})	0.5 (GC)	[6]
$Mn_{0.73}Ru_{0.27}O_{2-\delta}$	0.5 M H_2SO_4	η_{10} = 208	10 h (10 A cm^{-2})	0.28 (GC)	[7]
RuCo@CD	0.5 M H_2SO_4	η_{10} = 190	20 h (10 A cm^{-2})	0.905 (GC)	[8]
Rh-RuO_2	0.5 M H_2SO_4	η_{10} = 161	700 h (50 A cm^{-2})	1.25 (Ti Mesh)	[9]
RuO_2/D-TiO_2	0.5 M H_2SO_4	η_{10} = 180	100 h (10 mA cm^{-2})	5.0 (Carbon Paper)	[10]
**$Re_{0.1}Ru_{0.9}O_2$**	**0.1 M $HClO_4$**	**η_{10} = 199**	**300 h (10 mA cm^{-2})**	**1.0 (Carbon Paper)**	**This work**

Table S3. The fitting results of Ru 3$p_{3/2}$ spectra from XPS of post-catalysts.

samples	Ru^{4+}		Ru^{3+}		Ru^{4+}/Ru^{3+}
	Position/eV	Area	Position/eV	Area	
$Re_{0.1}Ru_{0.9}O_2$	462.5	57576.2	465.5	17176.2	3.4
HM–RuO_2	462.5	59882.1	465.5	13882.1	4.3

References

[1] Wu, Y.; Yao, R.; Zhao, Q.; Li, J.; Liu, G. La-RuO_2 Nanocrystals with Efficient Electrocatalytic Activity for Overall Water Splitting in Acidic Media: Synergistic Effect of La Doping and Oxygen Vacancy. *Chem. Eng. J.* **2022**, *439*, 135699.

[2] Liu, C.X.; Jiang, Y.B.; Wang, T.; Li, Q.S.; Liu, Y.Z. Nano Si-doped Ruthenium Oxide Particles from Caged Precursors for High-performance Acidic Oxygen Evolution. *Adv. Sci.* **2023**, *10*, 2207429.

[3] Sun, S.C.; Jiang, H.; Chen, Z.Y.; Chen, Q.; Ma, M.Y.; Zhen, L.; Song, B.; Xu, C.Y. Bifunctional WC-supported RuO_2 Nanoparticles for Robust Water Splitting in Acidic Media. *Angew. Chem. Int. Ed.* **2022**, *61*, 202202519.

[4] Wu, Z.-Y.; Chen, F.-Y.; Li, B.; Yu, S.-W.; Finfrock, Y.Z.; Meira, D.M.; Yan, Q.-Q.; Zhu, P.; Chen, M.-X.; Song, T.-W.; et al. Non-iridium-based Electrocatalyst for Durable Acidic Oxygen Evolution Reaction in Proton Exchange Membrane Water Electrolysis. *Nat. Mater.* **2022**, *22*, 100-108.

[5] Hou, L.Q.; Li, Z.J.; Jang, H.; Wang, Y.; Cui, X.M.; Gu, X.M.; Kim, M.G.; Feng, L.G.; Liu, S.G.; Liu, X. Electronic and Lattice Engineering of Ruthenium Oxide towards Highly Active and Stable Water Splitting. *Adv. Energy Mater.* **2023**, *13*, 2300177.

[6] Huang, K.; Lin, C.L.; Yu, G.Q.; Du, P.; Xie, X.Y.; He, X.; Zheng, Z.C.; Sun, N.; Tang, H.L.; Li, X.B.; et al. Ru/Se-RuO_2 Composites via Controlled Selenization Strategy for Enhanced Acidic Oxygen Evolution. *Adv. Funct. Mater.* **2023**, *33*, 2211102.

[7] Wang, K.X.; Wang, Y.L.; Yang, B.; Li, Z.J.; Qin, X.T.; Zhang, Q.H.; Lei, L.C.; Qiu, M.; Wu, G.; Hou, Y. Highly Active Ruthenium Sites Stabilized by Modulating Electron-feeding for Sustainable Acidic Oxygen-evolution Electrocatalysis. *Energy Environ. Sci.* **2022**, *15*, 2356-2365.

[8] Feng, T.L.; Yu, G.T.; Tao, S.Y.; Zhu, S.J.; Ku, R.Q.; Zhang, R.; Zeng, Q.S.; Yang, M.X.; Chen, Y.X.; Chen, W.H.; et al. A Highly Efficient Overall Water Splitting Ruthenium-cobalt Alloy Electrocatalyst across a Wide pH Range Electronic Coupling with Carbon Dots. *J. Mater. Chem. A* **2020**, *8*, 9638-9645.

[9] Wang, Y.; Yang, R.; Ding, Y.; Zhang, B.; Li, H.; Bai, B.; Li, M.; Cui, Y.; Xiao, J.; Wu, Z.-S. Unraveling Oxygen Vacancy Site Mechanism of Rh-doped RuO_2 Catalyst for Long-lasting Acidic Water Oxidation. *Nat. Commun.* **2023**, *14*, 1412.

[10] Wang, X.; Wan, X.; Qin, X.; Chen, C.; Qian, X.; Guo, Y.; Xu, Q.; Cai, W.-B.; Yang, H.; Jiang, K. Electronic Structure Modulation of RuO_2 by TiO_2 Enriched with Oxygen Vacancies to Boost Acidic O_2 Evolution. *ACS Catal.* **2022**, *12*, 9437-9445.
